# Lobe Specific Ca^2+^-Calmodulin Nano-Domain in Neuronal Spines: A Single Molecule Level Analysis

**DOI:** 10.1371/journal.pcbi.1000987

**Published:** 2010-11-11

**Authors:** Yoshihisa Kubota, M. Neal Waxham

**Affiliations:** Department of Neurobiology and Anatomy, University of Texas Medical School, Houston, Texas, United States of America; University of Auckland, New Zealand

## Abstract

Calmodulin (CaM) is a ubiquitous Ca^2+^ buffer and second messenger that affects cellular function as diverse as cardiac excitability, synaptic plasticity, and gene transcription. In CA1 pyramidal neurons, CaM regulates two opposing Ca^2+^-dependent processes that underlie memory formation: long-term potentiation (LTP) and long-term depression (LTD). Induction of LTP and LTD require activation of Ca^2+^-CaM-dependent enzymes: Ca^2+^/CaM-dependent kinase II (CaMKII) and calcineurin, respectively. Yet, it remains unclear as to how Ca^2+^ and CaM produce these two opposing effects, LTP and LTD. CaM binds 4 Ca^2+^ ions: two in its N-terminal lobe and two in its C-terminal lobe. Experimental studies have shown that the N- and C-terminal lobes of CaM have different binding kinetics toward Ca^2+^ and its downstream targets. This may suggest that each lobe of CaM differentially responds to Ca^2+^ signal patterns. Here, we use a novel event-driven particle-based Monte Carlo simulation and statistical point pattern analysis to explore the spatial and temporal dynamics of lobe-specific Ca^2+^-CaM interaction at the single molecule level. We show that the N-lobe of CaM, but not the C-lobe, exhibits a nano-scale domain of activation that is highly sensitive to the location of Ca^2+^ channels, and to the microscopic injection rate of Ca^2+^ ions. We also demonstrate that Ca^2+^ saturation takes place via two different pathways depending on the Ca^2+^ injection rate, one dominated by the N-terminal lobe, and the other one by the C-terminal lobe. Taken together, these results suggest that the two lobes of CaM function as distinct Ca^2+^ sensors that can differentially transduce Ca^2+^ influx to downstream targets. We discuss a possible role of the N-terminal lobe-specific Ca^2+^-CaM nano-domain in CaMKII activation required for the induction of synaptic plasticity.

## Author Summary

Calmodulin is a versatile Ca^2+^ signal mediator and a buffer in a wide variety of body organs including the heart and brain. In the brain, calmodulin regulates intracellular molecular processes that change the strength of connectivity between neurons, thus contributing to various brain functions including memory formation. The exact molecular mechanism as to how calmodulin regulates these processes is not yet known. Interestingly, in other excitable tissues, including the heart, each of two lobes of calmodulin responds differentially toward Ca^2+^ influx and toward its target molecules (e.g., ion channels). This way, calmodulin precisely controls the Ca^2+^ dynamics of the cell. We wish to test if a similar mechanism may be operational in neurons so that two lobes of calmodulin interact differentially with Ca^2+^ ions to activate different downstream molecules that control the strength of connections between neurons. We constructed a detailed simulation of calmodulin that allows us to keep track of its interactions with Ca^2+^ ions and target proteins at the single molecule level. The simulation predicts that two lobes of calmodulin respond differentially to Ca^2+^ influx both in space and in time. This work opens a door to future experimental testing of the lobe-specific control of neural function by calmodulin.

## Introduction

Calmodulin (CaM) is a ubiquitous Ca^2+^ buffer and signaling molecule in cells. In the excitatory synapse of hippocampal CA1 pyramidal neurons, the activation of CaM dependent enzymes results in the induction of synaptic plasticity (e.g., long-term potentiation (LTP) and long-term depression (LTD)) [1]. The induction of NMDA receptor dependent LTP and LTD require increased Ca^2+^ and subsequent activation of CaM-dependent downstream enzymes: CaM-dependent protein kinase II (CaMKII) and calcineurin. Injection of CA1 pyramidal cells with peptides that block CaMKII activity inhibited the induction [2], [3], but not maintenance [4] of LTP, while injection of the activated form of the enzyme also produced LTP-like plasticity [5], [6]. LTD is also critically dependent on Ca^2+^ and it appears that the CaM-dependent phosphatase, protein phosphatase 2B (calcineurin) is involved in LTD induction [7]. The simplest correlative explanation for these results is that LTD is induced by intermediate levels of Ca^2+^ that activate CaM and subsequently calcineurin but not CaMKII. Conversely, higher levels of Ca^2+^ initiate CaM-dependent CaMKII activation and autophosphorylation, leading to LTP induction. However, it is still unknown how Ca^2+^ and CaM regulate two opposing processes as distinct as LTP or LTD in such a precise and controlled manner.

Besides being a major signaling molecule, CaM also functions as a primary Ca^2+^ buffer in CA1 pyramidal neurons [8]. In fact, most CA1 pyramidal neurons contain CaM but not other EF-hand Ca^2+^ binding proteins (e.g., parvalbumin and calretinin) (reviewed in [9]). An exception is calbindin-D28K, which is expressed in a subpopulation of CA1 pyramidal neurons but only in rat ([10], [11]). CaM binds four Ca^2+^ ions, two in its N-terminal lobe and two in its C-terminal lobe [12]. The binding sites in the N-terminal lobe are lower affinity [13] but exhibit faster kinetics as opposed to the higher affinity, slower kinetics of the C-terminal lobe sites [14], [15]. Surprisingly little is known as to how such a protein with multiple Ca^2+^ binding sites influences the diffusion of Ca^2+^ in the cell. Most pre-existing theories of Ca^2+^ binding and diffusion assume a fast binding of Ca^2+^ and single Ca^2+^ binding site for the buffer (see reviews by [16]). In addition, recent experimental data suggest that each lobe of CaM has different affinity toward its downstream target (CaMKII and calcineurin) [17], [18], [19]. As each lobe differentially responds to Ca^2+^ signals and downstream targets, it is possible that these lobe specific properties play distinct biological roles in synaptic spines (see Discussion for more details). This motivated us to dissect the spatial-temporal dynamics of lobe specific Ca^2+^-CaM interaction in detail at the single molecule level.

Many elegant experimental measurements have been made of dendritic spine Ca^2+^ [20], [21], [22], [23], [24]. These measurements largely rely on a spatially averaged Ca^2+^ signal generated from fluorescence imaging of dyes whose quantum efficiency changes upon Ca^2+^ binding. As such, they contain no direct information relative to the issue of possible micro- or nano-domains of intracellular Ca^2+^. The problem is exacerbated by the high diffusion coefficients of free and dye bound Ca^2+^ which additionally smears the spatial signal in time frames relevant for Ca^2+^-imaging experiments. These and other caveats related to dye-based Ca^2+^-imaging experiments were recently reviewed [25]. In addition, we do not have an effective fluorescence reporter to detect and monitor Ca^2+^ binding to each lobe of CaM at the single molecule level. As such, mathematical models and computer simulations are presently the only tractable means of investigating this critical aspect of synaptic physiology. Furthermore, in a medium size dendritic spine (i.e., sphere-shaped spine head of 500 nm diameter), the concentration of 1 µM of any chemical species corresponds to ∼40 molecules. The basal (resting) level of spine Ca^2+^ is 50∼100 nM which corresponds to 2∼4 molecules of Ca^2+^ ions. Under such a circumstance, the behavior of single molecules within synaptic spines is not well described by the concentration-based mathematical approach such as reaction diffusion equation.

Here we report the single molecule level analysis of Ca^2+^-CaM interaction within a dendritic spine using a novel particle-based event-driven Monte Carlo algorithm, which we call Cellular Dynamics Simulator (CDS, [26]). Unlike other commonly used Monte Carlo simulation (e.g., MCell, [27]), it explicitly takes account of volume exclusion and collision between diffusing molecules in order to accurately simulate chemical reactions in the cellular interior. Using this simulator and first passage time theory, we dissect the mechanisms that influence the dynamics of Ca^2+^-CaM interaction at the single molecule level. We use a model of CaM built upon detailed kinetic data and ask if the lobe specific spatial-temporal micro-domain of Ca^2+^-CaM activation *can* exist and if so how it is *biophysically* regulated in a small sub-cellular compartment like dendritic spines. We employ a statistical spatial point pattern analysis [28] to understand the spatial profile of Ca^2+^-CaM interactions. The combination of spatial point pattern analysis and particle based Monte Carlo simulation is a unique computational strategy used in this study. Our analysis shows a higher sensitivity of the N-terminal lobe to the location and influx rate of Ca^2+^ from typical receptor/channel sources. Each lobe of CaM functions as distinct Ca^2+^ sensors and responds differentially to Ca^2+^ influx both in space and in time. Coupled with the experimental knowledge that different enzymes bind preferentially to either the N- or C-lobes of Ca^2+^ saturated CaM, we propose a possible explanation for how two opposing Ca^2+^/CaM-dependent enzymes can be differentially activated.

## Results

### The Importance of Chemical Kinetics: Slow and Fast Ca^2+^ Binding to CaM and the First Passage Time Analysis

**Figure 1:**
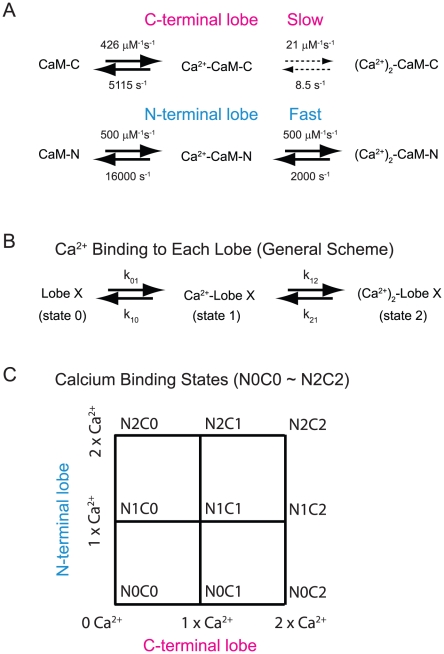
Kinetic diagrams showing the interactions between Ca^2+^ and each lobe of CaM. (A) CaM binds four Ca^2+^ ions, two on the C-terminal lobe (upper diagram), and two on the N-terminal lobe (lower diagram). Each arrow in the panel identifies the corresponding rate constant. The upper rightward arrows indicate the Ca^2+^ association rate and the lower leftward arrows are the Ca^2+^ dissociation rates. These values are taken from [30]. Note the slow Ca^2+^ association rate for the second Ca^2+^ binding step for the C-terminal lobe. (B) To explain the mathematical formulation in Fig. 2 (Eq. 1∼3), here we present a generalized reaction scheme for Ca^2+^ binding to each lobe of CaM. Compare theoretical formulas (Eq. 1∼3) with the symbolic notations in Panel B. (C) Each lobe of CaM has three different Ca^2+^ binding states (i.e., 0, 1, and 2 Ca^2+^ bound states, N0∼N2 and C0∼C2). CaM therefore has 3×3 = 9 different Ca^2+^ binding states. The notation shown here will be used throughout this work (e.g., Fig. 7).

Fig. 1A illustrates the Ca^2+^ binding and unbinding pathway for each lobe of CaM. As shown, Ca^2+^ binding to the N-terminal lobe and the first Ca^2+^ binding event to the C-terminal lobe are diffusion limited while the second Ca^2+^ binding to the C-terminal lobe is the rate-limiting step in achieving the fully Ca^2+^-saturated state. If this Ca^2+^ binding step at the C-terminal lobe is much slower than the diffusion of Ca^2+^, the majority of Ca^2+^ ions that entered the spine head will have moved away from the channel without saturating local CaM molecules. The spatial profile of the C-terminal lobe or full Ca^2+^ saturation of CaM may then be less sensitive to the location of Ca^2+^ channels. On the other hand, if the N-terminal lobe Ca^2+^ saturation is fast as compared to the Ca^2+^ diffusion, its Ca^2+^ saturation may be more closely localized to the Ca^2+^ channels. Thus, three biophysical factors become important in understanding the spatial domain of Ca^2+^-CaM interactions. The first is how fast each lobe of CaM becomes Ca^2+^ saturated with a given concentration of Ca^2+^. The second is how fast Ca^2+^ ions escape from the spine. The third is how steep or flat the gradient of Ca^2+^ ion distribution will be in the spine head with a given Ca^2+^ injection rate through Ca^2+^ channels.

In this section, we analyze the first biophysical factor, which we call the (mean) first passage time: the (average) length of transition time required for each lobe of CaM molecule to reach the Ca^2+^ saturated state from a basal (apo-) state. In fact, a mathematical formula is already available to calculate this mean first passage time (Equations 5, 29 in [29]). In their single molecule biophysical analysis, Shaevitz et al. [29] used an algebraic recursive method to derive the Laplace transform of the first passage time distribution. Fig. 1B and Eq. 1∼2 explain their formalism applied to Ca^2+^-CaM interactions. Here we define State “0” as a Ca^2+^ free (apo) form, State “1” as one Ca^2+^ ion bound form, and State “2” as a two Ca^2+^ ion bound form of a given lobe. The symbols k^X^ *_ij_* in Fig. 1B denotes the rate constant between State *i* and State *j* (*i, j = 0, 1, 2*) of lobe X ( = N or C). Thus, each lobe has three states and the whole CaM molecule has nine states (Fig. 1C).

The resultant Laplace transform 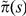 of the distribution of first passage time 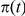 is:
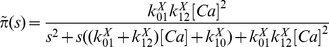
(1)where [Ca] is the given concentration of Ca^2+^ (Note, in order to apply Eq. 29 in [29], we needed to multiply the association rate constant by the concentration of Ca^2+^). Here we assume the system is well-stirred and the concentration of Ca^2+^ is constant (time-invariant). Then, the mean first passage time (<*t*>) can easily be found through differentiation (see Eq. 5 in [29]):
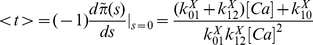
(2)Note that the dissociation rate (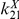) of the second Ca^2+^ is not included in the formula. The latter rate determines the lifetime of fully Ca^2+^ saturated state of each lobe but it does not influence the first passage time. Therefore, three kinetic rates (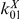, 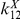, 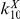) and Ca^2+^ concentration determine the lobe specific first passage time. Note that both lobes have similar association rates for the first Ca^2+^ ions (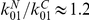) (Fig. 1A). The difference in the second Ca^2+^ binding rates (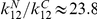) is large as compared to the dissociation of the first Ca^2+^ ion (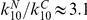) (Fig. 1A). Thus, in Eq. 2, the second Ca^2+^ binding rates (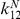, 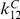) determine the difference of the first passage time between the N- and C-lobes.

**Figure 2:**
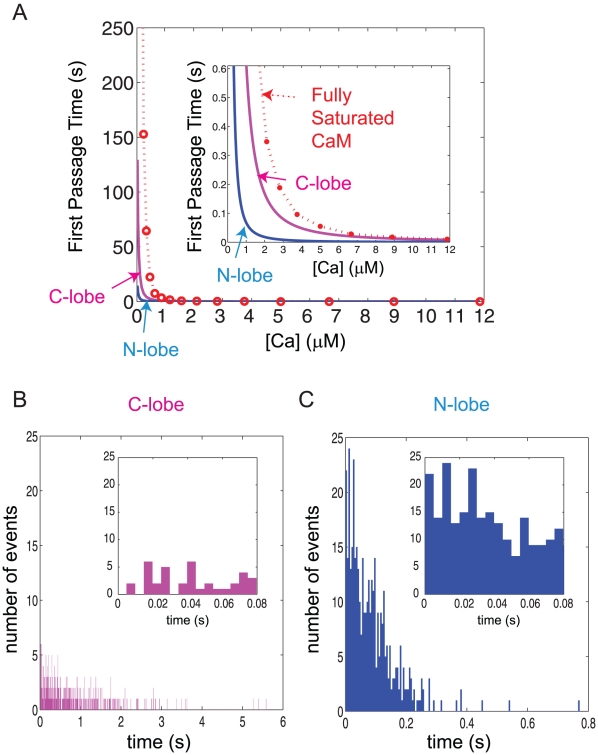
Mean first passage time of Ca^2+^ binding to CaM. (A) The mean transition time of each lobe of CaM going from the basal (apo-) state to the two Ca^2+^ bound state and fully Ca^2+^ saturated CaM is displayed as a function of the Ca^2+^ concentration (blue and magenta, for the N-terminal lobe and C-terminal lobe. We used the numerical simulation to calculate the mean first passage time for full Ca^2+^ saturation of CaM (indicated by the red circles). Note the unit of first passage time (y-axis) is seconds. The range of Ca^2+^ concentrations considered here is from 0.05 µM (resting level) to ∼12 µM (close to the peak free Ca^2+^ concentration during synaptic stimulation). The inset shows the expanded scale of mean first passage time near ∼1 µM Ca^2+^ concentrations. (B) and (C) The first passage time distribution taken from a single stochastic simulation run of 400 CaM molecules with 1 µM of Ca^2+^. Panel B is the C-terminal lobe and Panel C is the N-terminal lobe Ca^2+^ saturation, respectively. We used the same bin size (5 ms) to plot the first passage histogram for both lobes. The insets are the enlarged view of the histograms up to 80 ms (close to the duration of the NMDA receptor Ca^2+^ transient) showing that much larger numbers of the N-terminal lobe are Ca^2+^ saturated than the C-terminal lobe.

Fig. 2A is a numerical display of this formula showing that the first passage time sharply increases as we decrease the Ca^2+^ concentration (the unit of time, y-axis, is in seconds). As predicted, the mean first passage time for the C-terminal lobe (magenta) is much longer than the N-lobe (blue). For comparison, we show the first passage time for full Ca^2+^ saturation of CaM; the mean first passage time to reach the state N2C2 in Fig. 1C. As one can see from the diagram in Fig. 1C, this first passage time depends on all Ca^2+^ association and dissociation pathways for both lobes and is influenced by the lifetime of the Ca^2+^ saturated states of each lobe. The corresponding mathematical formula will be much more complicated than Eq. 1 and 2 and therefore, we calculated this quantity numerically using an extended version of the Gillespie type stochastic algorithm (see [8], [30] for more details). The results presented in Fig. 2A suggest that the N-terminal lobe may respond to a short Ca^2+^ transient but the C-terminal lobe may not if the transient is shorter than the first passage time of C-lobe Ca^2+^ saturation. For example, NMDA receptor type Ca^2+^ transients (∼1 µM peak with duration of ∼80–200 ms) may not result in significant CaM saturation in the spine. In fact, at a ∼1 µM Ca^2+^ concentration, the mean first passage time for the C-terminal lobe (or full Ca^2+^ saturation of CaM) is much longer than the duration of the Ca^2+^ transient (Fig. 2A upper right inset).

Such straightforward interpretation of the first passage time analysis, however, could be misleading. Note that we have only discussed the mean but not the entire distribution (or standard deviation) of the first passage time. In addition, we ignored the fact that the number of Ca^2+^ ions may be limited in the dendritic spines and that their concentration is not constant as postulated in Eq. 1∼2: the N-terminal lobe and the C-terminal lobes on the same or different CaM molecules will compete for the limited number of Ca^2+^ ions. As for the stochastic fluctuation, we can derive the standard deviation of the first passage time using the same analytic method described above:
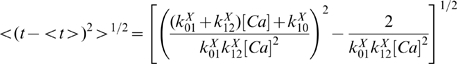
(3)The resultant standard deviation is very close to the mean first passage time for all Ca^2+^ concentrations (i.e., the coefficient of variation is >0.9 for all [Ca^2+^]<10 µM). The second term in the right-hand side of Eq. 3 (
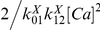
) is small because the first Ca^2+^ binding rate (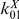) for both lobes are high and therefore the ratio of the right-hand sides of Eq. 3 and Eq. 2 approaches 1.

Fig. 2B and C show the histograms of the first passage time distribution for the N-terminal lobe (blue) and the C-terminal lobe (magenta) Ca^2+^ saturation, respectively, taken from a single stochastic simulation (the same bin size, 5ms, for both lobes and the total number of CaM molecules is 400). Fig. 2B clearly shows that the Ca^2+^ saturation of the C-terminal lobe is possible even if the mean first passage time is shorter than that of Ca^2+^ transient. However, the inset of Fig. 2B and 2C, i.e., the histogram up to 80 ms, predict that the N-terminal lobe Ca^2+^ saturation predominates and precedes that of the C-terminal lobe during the short Ca^2+^ transient. Knowing that two lobes of CaM compete for the limited amount of available Ca^2+^ ions in the dendritic spines, we predict that the N-terminal dominance for the short Ca^2+^ transient is more prominent in neurons.

This type of analysis, however, is further complicated when taking into account the non-homogeneous spatial distribution of molecules. When Ca^2+^ ions enter the spine head through a Ca^2+^ channel, a steep spatial gradient of Ca^2+^may be formed around the channel mouth (depending on the Ca^2+^ injection rate). At a single molecule level, it is the transient local (microscopic) “concentration” of Ca^2+^ (i.e., the number of Ca^2+^ collision events) felt by a CaM molecule that determines the probability of Ca^2+^ saturation of a given lobe of each CaM molecule. A CaM molecule can experience much higher (local) Ca^2+^ “concentration” than indicated by the bulk Ca^2+^ transient depending on its location with respect to the Ca^2+^ source.

The present work aims to describe a detailed analysis of this spatial stochastic phenomenon. However, before going into the detailed simulations, it is necessary to dissect each of the biophysical factors that we discussed at the beginning of this section. The last two of these factors determine the space- and time- dependent Ca^2+^ profile in the spines. Without such a systematic dissection, the interpretation of simulation results when trying to determine the spatial/temporal profile of CaM activation would not be possible. We next explored how fast Ca^2+^ ions escape from the spine.

### Escape Rate of Ca^2+^ from the Spines

**Figure 3:**
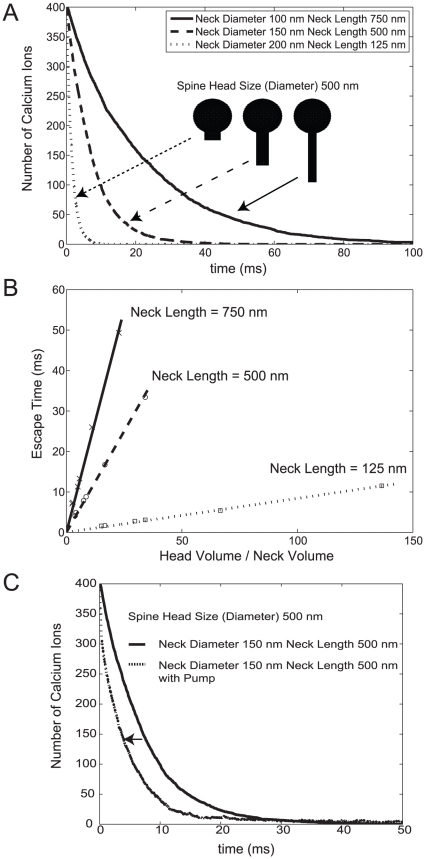
Narrow escape time of Ca^2+^ from CA1 spine. (A) We placed 400 Ca^2+^ ions randomly in the spine head compartment and let them diffuse out of the spine. Ca^2+^ ions are absorbed at the dendrite-spine boundary. Time course of Ca^2+^ decay form the spine compartment (head and neck) is shown for different geometries: short and wide neck (left), average/intermediate neck (center), long and think neck (right). The data is an average of 100 simulation runs. The decay process is well fit by a single exponential curve as predicted by Eq. 4 for all CA1 spine morphologies tested in this work (see the discussion in the main text). (B) We systematically varied the morphology of the spine within the known variation [70]. For each of these morphologies, we carried out the simulation as in Panel A and calculated the narrow escape time constant. The resultant narrow escape constants were plotted against the volume ratio of spine head and neck (V_h_/V_n_, x-axis). The unit of escape time (y-axis) is ms. (C) The narrow escape dynamics with (dashed black line) and without (solid black line) pumps. We placed Ca^2+^ pumps (PMCA and NCX.NCX) on the standard morphology spine (the center in Panel A) at a relatively high density to see the maximum impacts of pumps on Ca^2+^ narrow escape process.

The second factor that will determine the spatial profile of CaM activation is the escape rate of Ca^2+^ from the spine. Ca^2+^ ions that enter the spine through ion channels will eventually diffuse into the dendrites or be extruded by the Ca^2+^ pumps [23]. Here we focus on the impact of spine geometry and Ca^2+^ pumps on the escape rate of Ca^2+^ from the spines. We carry out this analysis in a stepwise manner. We first analyze the escape of Ca^2+^ via pure diffusion without Ca^2+^ pumps (or buffers) and establish the impact of spine morphology on the Ca^2+^ escape rate (Fig. 3A and B). Then we add Ca^2+^ pumps to examine their impact (Fig. 3C). This way we can isolate and understand the contribution of each of these factors in the regulation of the Ca^2+^ escape rate. In neurons, Ca^2+^ buffers such as CaM also influence this escape rate but in a highly complicated manner. We will study the effect of Ca^2+^ binding proteins (CaM) in the later sections when we combine all known biophysical factors in the detailed simulations.

Fig. 3A shows the time courses of Ca^2+^ decay for three different spine neck geometries. Here, we randomly placed a fixed number of Ca^2+^ ions ( = 400 that corresponds to ∼10 µM) in the head of a spherical spine and let them diffuse out of the spine to the dendrite. The diffusion coefficient (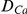) of Ca^2+^ was set to 200∼225 µm^2^/s (nm^2^/µs) [31]. Each curve in Fig. 3A represents the average of 100 simulation runs. Clearly, the longer and the narrower the neck, the slower the Ca^2+^ decay process. This is a so-called narrow escape problem and has been extensively investigated [32], [33]. As predicted by these theoretical studies, the simulated Ca^2+^ decay transient is well approximated by a single exponential decay term. These decay time constants fit well (the relative error <5%) with one of the pre-existing mathematical formula (the left-hand side of Eq. 4 below):
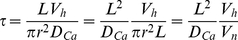
(4)where 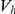, 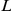, 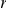 and 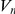 are the volume of the spine head, the length and the radius of spine neck, and the volume of neck, respectively [32]. Fig. 3B summaries our simulation results for different spine geometries. We plot the narrow escape time (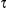) against the ratio of spine head and neck volume (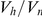) (x-axis). As shown all data points are aligned on straight lines, indicating that the narrow escape time is a linear function of the volume ratio (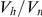) (see the right-hand side of Eq. 4). Note that Eq. 4 was previously tested against experimental data of molecular diffusion (using photo-bleaching recovery of fluorescein-dextran and enhanced green fluorescent protein) across spine-dendrite junctions in CA1 neurons [21], [24]. In other words, Eq. 4 is consistent with escape of diffusing molecules from real spines on CA1 neurons. Additional simulations confirm that Eq. 4 fits well with real spines when morphologies from 3D EM reconstructions are used (http://synapses.clm.utexas.edu/) (data not shown).

Another biophysical factor that regulates the Ca^2+^ decay from spines is Ca^2+^ pumps [20], [23]. The main Ca^2+^ extrusion mechanisms in CA1 spines are Na^+^/Ca^2+^ exchangers (NCX, NCKX) and plasma membrane Ca^2+^ ATPase (PMCA) [20], [34]. We have modeled both of them using the kinetic scheme used in [35] (see Methods for more details). Fig. 3C shows a Ca^2+^ clearance process with standard spine morphology (500 nm spine head diameter, 500 nm spine neck length and 150 nm spine neck diameter) with (dashed black line) and without pumps (solid black line). The fast decay time constant of Ca^2+^ in the presence of pump is ∼45% of the narrow escape time without pumps (∼5–6 ms). In this analysis, we have included NCX/NCKX and PMCA at the concentration close to the highest level known in the literature to examine the maximal impact that Ca^2+^ pumps would have on Ca^2+^ clearance. The Ca^2+^ transients with reduced number of pumps lie between the dashed and solid lines (data not shown).

Overall, the analyses in Fig. 3 show that the narrow escape time of Ca^2+^ without buffers in a standard spine in the presence of pumps is ∼5 ms or shorter. In the subsequent section, we will show that a major Ca^2+^ buffer in CA1 pyramidal neurons (i.e., CaM) slows down the Ca^2+^ decay to ∼10∼20 ms (the latter is close to that observed in the Ca^2+^ imaging analyses [20]. It is this brief time window that each lobe of CaM becomes Ca^2+^ saturated or not during each Ca^2+^ spike. The first passage time becomes a critical factor to understand the spatial profile of Ca^2+^-CaM interactions.

### Spatial Domains of the Ca^2+^ Signal in Dendritic Spines: The Critical Impact of Ca^2+^ Injection Rates

**Figure 4:**
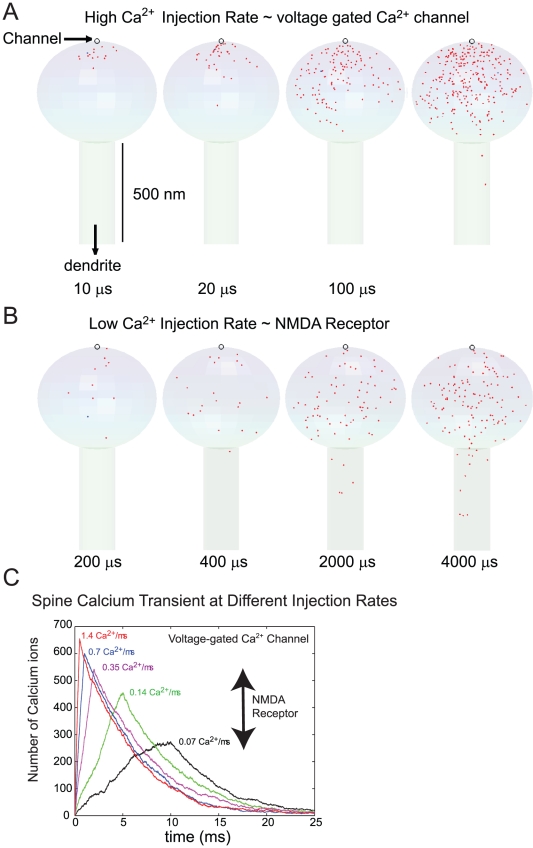
Differential profile of spatial Ca^2+^ domains in the dendritic spine. (A and B) A single Ca^2+^ channel was placed on the top of a “standard” sphere-headed spine (250 nm radius head attached to the cylindrical spine neck (75 nm radius and 500 nm length; the vertical scale bar = 500 nm). A total of 700 Ca^2+^ ions were injected at different rates and the spatiotemporal patterns of Ca^2+^ ion distribution (red points) were analyzed by a particle-based Monte Carlo simulation. The Ca^2+^ injection rates in Panel A and B were 1.4 and 0.07 Ca^2+^ ion per microsecond, respectively. (C) Summary data showing a plot of the total number of Ca^2+^ ions over the course of simulation (25 ms) for different Ca^2+^ injection rates as indicated. Curves were taken from a single simulation run for each Ca^2+^ injection rate. The range of Ca^2+^ injection rates for a NMDA type receptor is illustrated with a two headed black arrow. The maximum injection rate of 1.4 Ca^2+^/µs is representative of that through a voltage-gated Ca^2+^-channel.

Having established the impact of spine geometry on the Ca^2+^ extrusion process, we now analyze the third biophysical factor that influences the spatial gradient of spine Ca^2+^: the Ca^2+^ injection rate of channels. Since the kinetics of the voltage-gated Ca^2+^ channels and NMDA receptors are highly complicated, we used a “model stochastic Ca^2+^ channel” in this section. A single stochastic Ca^2+^ channel was placed on the top of the head of a standard spine (black circle in Fig. 4A; see Fig. 3C for the standard morphology of CA1 dendritic spine). This channel injects Ca^2+^ at a given (average) rate and we examine the relation between the Ca^2+^ injection rate and the spatio-temporal profile of Ca^2+^ transients in the spine. To realize the impact of Ca^2+^ injection rate in isolation on the spatiotemporal Ca^2+^ profile, there are no pumps or Ca^2+^ binding buffers in this model spine. Once injected, Ca^2+^ ions travel via simple diffusion until they are absorbed from the compartment at the spine-dendrite boundary (see the vertical arrow in Fig. 4A). We varied the rate, but the total number of injected Ca^2+^ ions was set to 700 so that the peak Ca^2+^ concentration would be in a physiological range (∼6–16 µM, i.e., ∼250–650 Ca^2+^ ions; see panel C of Fig. 4). Note these numbers are taken from the lowest estimated Ca^2+^ injection rate of NMDA receptors and the higher Ca^2+^ injection rates of voltage gated Ca^2+^ channels ([36], [37], [38]).

Fig. 4 Panel A and B show the location of Ca^2+^ ions (not to scale) at designated time points after the start of Ca^2+^ injection. The mean Ca^2+^ injection rates in Panel A and B are 1.4 and 0.07 Ca^2+^ ions per microsecond, respectively. At the higher injection rate (1.4 ions/µs), there is a build-up of Ca^2+^ ions near the channel (Panel A) while such a build up is not evident in Panel B. Note that the time points chosen for Panel A and B are 20-fold different so that the total number of Ca^2+^ injected by the indicated time points in Panel A (10, 20, 100, 200 µs) and B (200, 400, 2000, 4000 µs) are identical. Ca^2+^ ions can travel ∼140 nm from the channel via diffusion before the next Ca^2+^ ion exits the channel at injection rate of 0.07 ions/µs. At a higher Ca^2+^ injection rate, Ca^2+^ ions will accumulate near the channel pore before they diffuse away (red in Fig. 4C). As anticipated, the lower Ca^2+^ injection rate (black) leads to a much lower peak Ca^2+^ number (concentration) than the higher Ca^2+^ injection rates. Ca^2+^ ion can travel more than 1 µm away from the channel during 1 ms. During a 10 ms Ca^2+^ injection period, a significant fraction of Ca^2+^ ions has already left the spine. Thus, we have lower Ca^2+^ peak than at the higher Ca^2+^ injection rate. After the peak, the Ca^2+^ level decreases with a time constant of ∼7–8 ms for all Ca^2+^ injection rates. This decay process is controlled by the diffusion and is consistent with the narrow escape rate we calculated in Fig. 3.

Fig. 4 clearly shows the impact of Ca^2+^ injection rates on the spatial and temporal dynamics of Ca^2+^ transients in dendritic spines. The relative lack of a Ca^2+^ gradient in Fig. 4B and the long first passage time of the C-terminal lobe of CaM in Fig. 2 suggest that a spatial gradient of the Ca^2+^-saturated C-terminal lobe may not form. However, as mentioned at the beginning of this section, we need to include CaM and examine the combined effect of all of these biophysical factors on the spatial profile of Ca^2+^-CaM interactions. The second half of Results provides this analysis.

### Combining Chemical Kinetics and Space: Spatial Domains of Lobe-Specific Ca^2+^-CaM Activation

In the previous sections, we studied the impact of three biophysical factors: the first passage time (Fig. 2), the narrow escape time (Fig. 3), and the impact of Ca^2+^ injection rate on the Ca^2+^ micro-domain (Fig. 4). In this section, we wish to study the combined effects of these factors on the spatial-temporal pattern of Ca^2+^-CaM interaction. As a first step, we placed a single “model Ca^2+^ channel” as in Fig. 4 but add CaM to assess the impact of Ca^2+^ injection rates on the Ca^2+^-CaM interaction. Besides the “artificial” model channel, we included CaM and Ca^2+^ pumps. We distributed 1600 molecules of CaM (i.e., 40 µM) uniformly within the spine volume (the estimated concentration of CaM in CA1 dendritic spines is 10∼100 µM, [8]). Before injecting Ca^2+^ ions the entire system is equilibrated at basal Ca^2+^ conditions, i.e., ∼40–46 Ca^2+^ bound CaM molecules with ∼2 free Ca^2+^ ions (the latter correspond to 50 nM of basal free Ca^2+^ concentration). At this basal condition, majority of CaM molecules are Ca^2+^ free or in a single Ca^2+^ bound form and none of their lobes are Ca^2+^ saturated. The diffusion coefficient of CaM varies between 2∼20 µm^2^/s (nm^2^/µs) [30], [39]. In this section, we set it to 20 µm^2^/s (nm^2^/µs) (but see our comments below).

**Figure 5:**
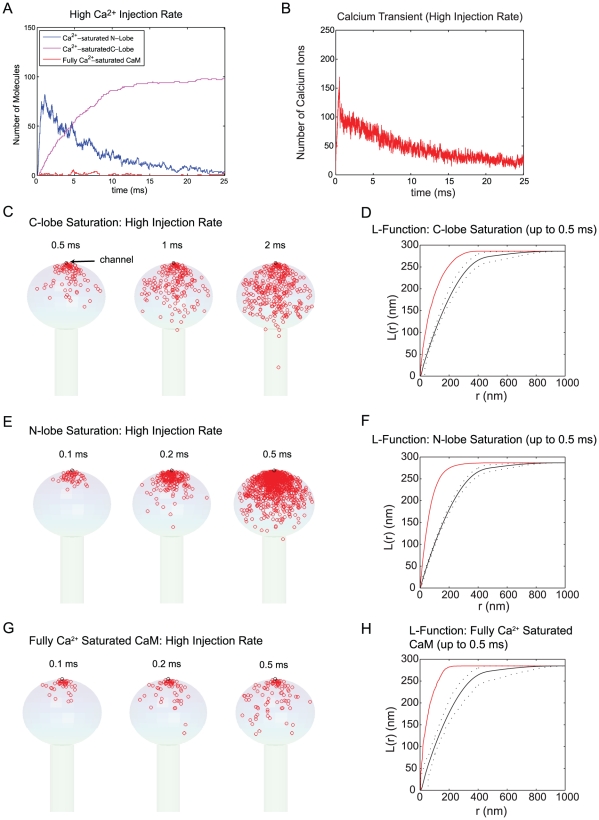
Spatial domain of Ca^2+^-CaM at high Ca^2+^ injection rates. 1600 CaM molecules (∼40 µM) were placed in the spine (head and neck). Ca^2+^ ions were injected through a single “model channel” placed on the top of spine head at a rate of 1.4 Ca^2+^/µs. The population dynamics of Ca^2+^ saturated N-lobes (blue) and C-lobes (magenta) lobe are depicted with fully Ca^2+^ saturated CaM (red; Panel A). The Ca^2+^ transient over the same 25 ms of simulation is also shown (Panel B). The location of Ca^2+^ saturation of C- and N- lobes of CaM and the fully Ca^2+^ saturated CaM are shown up to the designated time points (Panel C, E, and G, respectively). These results are collected from 15 simulation runs. We performed the statistical spatial point pattern analysis for the data in C, E, and G (see Results as well as Method sections for details). The mean, maximum, and minimum of Besag's L-function for complete spatial randomness were calculated (solid black line for the mean and dashed lines for max/min envelope) and compared with the L-function of the data points (up to the designated time point = 0.5 ms, in red) (Panel D, F, H).

The results in Fig. 5 show the dynamics of Ca^2+^/CaM with a channel of high Ca^2+^ injection rate (1.4 Ca^2+^ ions/µs and a total of 700 Ca^2+^ ions are injected as in Fig. 4). Fig. 5A shows the number of Ca^2+^ saturated N- and C-lobes (blue and magenta, respectively) and fully Ca^2+^-saturated CaM. The number of free Ca^2+^ ions in the spine is shown in Fig. 5B. Both Fig. 5A and 5B are taken from the same single simulation run. The result of stochastic simulation varies from one simulation run to the other; however, the overall qualitative dynamics in Fig. 5A and 5B are similar among different simulation runs. The N-terminal lobe of CaM binds Ca^2+^ much faster than the C-terminal lobe (Fig. 2). As a consequence, the number of Ca^2+^ saturated N-terminal lobes increases rapidly as Ca^2+^ is injected (blue line in Fig. 5A). After the termination of Ca^2+^ injection (at 500 µs), the N-terminal lobes quickly release Ca^2+^ and the C-terminal lobes slowly bind the available Ca^2+^ (Fig. 5A). Once bound, Ca^2+^ remains associated with the C-lobe for a relatively long time (the decay time constant is ∼120 ms) and the C-lobes therefore trap Ca^2+^ in the spine (Fig. 5A). The free Ca^2+^ level eventually returns to the basal level after a few hundred ms (data not shown). Another important point to note is that even at this high Ca^2+^ injection rate, the total number of fully Ca^2+^-saturated CaM molecule is less than ∼7. This number varies from simulation to simulation, but with a single Ca^2+^ channel, the number remains below 10 (over 100 simulation runs), a remarkably low number.

Panels C, E, and G of Fig. 5 show the spatial dynamics of each lobe of CaM taken from 15 simulation runs. During the early rising phase of their Ca^2+^ saturation, each lobe of CaM exhibits a nano-domain near the channel pore. For example, in Fig. 5E, we record the location (red circle) of each CaM molecule when its N-lobe becomes *first* Ca^2+^ saturated. We plot these accumulated locations of “first Ca^2+^ saturation event” up to the different designated time point in the figure (note each lobe may undergo multiple cycles of Ca^2+^ saturation, but only the first one is recorded in Panel C, E, and G in Fig. 5 and in subsequent figures). The formation of a Ca^2+^/CaM nano-domain is clear. A similar but less obvious nano-domain is observed for the C-terminal lobe (Panel C) and for the fully Ca^2+^-saturated CaM.

To further confirm these observations, we performed spatial point pattern analysis (see Methods and [28], [40], [41]). In this statistical analysis, we counted the number of the Ca^2+^ saturation events (e.g., as shown in Fig. 5E for the N-terminal lobe) and then randomly distributed the same number of points within the spine volume. We calculated a so-called (Besag's) L-function (see Methods for details) for this random point pattern. We repeated this process 1000 times and calculated the mean and the maximum and minimum envelope of the L-function (the black dotted lines in Fig. 5F) for the set of 1000 randomly generated spatial patterns. We then calculated the L-function for the original data point pattern of Ca^2+^ saturation and compared this (the red line in Fig. 5F) with that of complete spatial randomness (the black lines in Fig. 5F). The L-function of data (red) is outside of the maximum and minimum envelopes (black dotted lines) indicating that the given point pattern is *not* random. In this case, L-function is larger than the maximum envelope and it is typical of spatial clustering. We performed a similar analysis for the C-terminal lobe (Fig. 5D) and fully Ca^2+^ saturated CaM (Fig. 5H) and obtained the same conclusion (non-randomness). For all cases, we also performed (two-sample) Kolmogorov-Smirnov (goodness-of-fit hypothesis) test (significance level = 0.05) [28] to verify the conclusion of envelope test.

In summary, the high Ca^2+^ injection rate results in a transient Ca^2+^-CaM nano-domain (for both lobes of CaM). The N-terminal lobe responds to and senses the Ca^2+^ gradient much faster than the C-lobe (blue Fig. 5A). The C-lobe's response is resistant to the Ca^2+^ gradient because of its longer first passage time (i.e., slow binding kinetics of Ca^2+^). Note we recorded and analyzed only the *first* Ca^2+^ saturation events for each lobe of each CaM molecules. The relatively widespread C-terminal lobe Ca^2+^ saturation in Panel C, therefore, is *not* because the high affinity C-terminal lobe carries Ca^2+^ ions while diffusing away from the channel.

**Figure 6:**
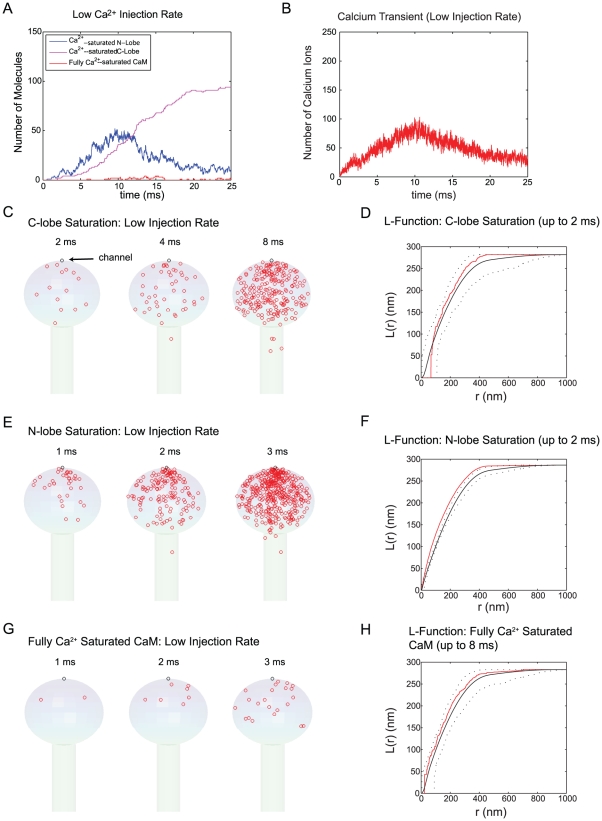
Spatial domain of Ca^2+^-CaM at low Ca^2+^ injection rates. Ca^2+^ ions were injected through a single “model channel” placed on the top of spine head at a rate of 0.07 Ca^2+^ ions per microsecond, 20-fold lower than that shown in Fig. 5. We populated the spine with the same number of CaM molecules (1600) as in Fig. 5. The population dynamics of Ca^2+^ saturated N-lobe (blue) and C-lobe (magenta) are depicted along with fully Ca^2+^ saturated CaM (red; Panel A). The Ca^2+^ transient over 25 ms of simulation is also shown (Panel B). The location of Ca^2+^ saturation of C- and N- lobes of CaM and the fully Ca^2+^ saturated CaM are shown up to the designated time points (Panel C, E, and G, respectively). These results are collected from 15 simulation runs. We performed the statistical spatial point pattern analysis for the data in C, E, and G (see Results as well as Method sections for details). The mean, maximum, and minimum of Besag's L-function for complete spatial randomness were calculated (solid black line for the mean and dashed lines for max/min envelope) and compared with the L-function of the data points (up to the designated time point = 2 ms for each lobe and 8 ms for fully Ca^2+^ saturated CaM, in red) (Panel D, F, H).

What if we reduce the Ca^2+^ injection rate? Fig. 4 indicates that the spatial gradient of Ca^2+^ is less prominent with a reduced Ca^2+^ injection rate. One possible scenario is that, under such a condition, only N-terminal lobe with higher Ca^2+^ binding kinetics (Fig. 2) can detect and sense the spatial gradient. The Ca^2+^ saturation of C-terminal lobe and/or full Ca^2+^ saturation of CaM may show relatively homogeneous spatial patterns under this condition. Fig. 6 shows results to test this prediction. The simulation conditions are the same as in Fig. 5 except the Ca^2+^ injection rate is reduced to 0.07 Ca^2+^ per microsecond. This is close to the lowest Ca^2+^ injection rate observed for a single NMDA receptor Ca^2+^ current [36], [37], [38]. Panel A and B in Fig. 6 show the population dynamics of Ca^2+^ saturated N- and C-terminal lobe, fully Ca^2+^ saturated CaM (A), and free Ca^2+^ ions (B). The difference in the rising phase of Ca^2+^ saturated N- and C- terminal lobes observed in Fig. 5A becomes less obvious at these lower rates of Ca^2+^ influx. The Ca^2+^ saturated N- and C-terminal lobes increase at a similar rate but the N-terminal lobe exhibits a larger fluctuation due to its fast Ca^2+^ dissociation rate. Again, the number of fully Ca^2+^ saturated molecules is small (less than 5∼10) over the course of a 25 ms simulation experiment.

In addition, the location of Ca^2+^ saturation for each lobe becomes less localized around the channel (Fig. 6C and 6E). It still looks like the N-lobe exhibits a nano-domain but it is unclear by a simple inspection of the data as to whether a nano-domain exists for the C-terminal lobe. Up to the time points 2 ms and 4 ms, the Ca^2+^ saturation of the C-terminal lobe takes place throughout the entire spine head. The distribution of these points appears to be random. To confirm whether this pattern is random or not, we carried out the same statistical analysis as that used in Fig. 5 (panel D, F, H). Clearly, the data point patterns in Panel D and F (red line) are closer to the maximum envelope (black dotted line) of complete spatial randomness but the N-terminal lobe data pattern shows a deviation from the complete spatial randomness. This result was again confirmed by Kolmogorov-Smirnov test. The spatial pattern of the C-terminal lobe and full Ca^2+^-CaM saturation lie within the maximum/minimum envelope and did not suggest significant deviations from the spatial randomness.

In conclusion, the N-terminal lobe exhibits a transient Ca^2+^-activated nano-domain at both lower and higher Ca^2+^ injection rates. This indicates that the kinetic property of the N-terminal lobe (Fig. 1 and 2) is the major determinant of the spatial pattern formation by the N-terminal lobe. In fact, we repeated simulations used to produce Figs. 5 and 6 with different spine morphologies (with shorter and longer spine neck as shown in Fig. 3A) and obtained similar results as to the N-terminal lobe specific nano-domain (Fig. S1 and Fig. S2). We also set the diffusion coefficient of CaM to 2 µm^2^/s (nm^2^/µs) and repeated simulations in Fig. 5 and 6 (Fig. S1 and Fig. S2). As long as CaM molecules are randomly distributed within the spine volume (at time 0), neither the diffusion coefficient nor the concentration of CaM (even when reduced to 10 µM) affected the high sensitivity of the N-terminal lobe to the Ca^2+^ influx. It appears that the Ca^2+^ binding kinetics of CaM (first passage time) is the major determinant of the lobe specific spatial pattern formation during Ca^2+^ influx.

In addition, the spatial pattern of fully Ca^2+^ saturated CaM was also influenced by the Ca^2+^ injection rate (Fig. 5A, 6A, 5H, and 6H). Recall that Ca^2+^ dissociation from the C-terminal lobe is slower than from the N-terminal lobe (Fig. 1A). The C-terminal lobe remains fully Ca^2+^ saturated for extended time (>100 ms) during which CaM (or any Brownian particle of the same diffusion coefficient) can travel a distance equal to or larger than the entire spine head volume. CaM can reach its fully Ca^2+^ saturated state when additional Ca^2+^ binds to the N-terminal lobe (note again, the *first* Ca^2+^ saturation event of the C-terminal lobe is less sensitive to the location of the Ca^2+^ source as compared to the N-terminal lobe). Alternatively, if Ca^2+^ injection rate is high and the transient Ca^2+^ concentration is adequate, CaM can reach the fully Ca^2+^ saturated state via N-terminal lobe Ca^2+^ saturation before Ca^2+^ saturates the C-terminal lobe because the first passage time for the N-terminal lobe is shorter than the C-terminal lobe (Fig. 2). The latter pathway may be responsible for the nano-domain of fully Ca^2+^ saturated CaM observed in Fig. 5G and Fig. 5H. If these two modes of Ca^2+^ saturation exist, they would have different physiological impacts of CaM signaling system as the two lobes of CaM have distinctive binding affinities for different targets. A detailed inspection of Fig. 5 and Fig. 6 simulation results in the next section reveals and confirms these two Ca^2+^ saturation pathways of CaM and their dependence on the Ca^2+^ injection rates.

### Single Molecule Level Analysis Reveals Distinctive Ca^2+^ Binding Pathways That Depend on the Ca^2+^ Injection Rate

**Figure 7:**
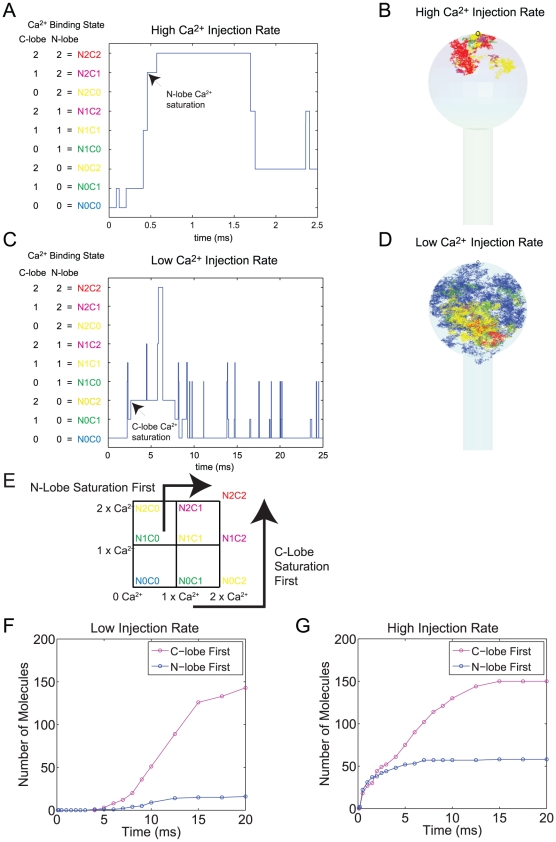
Single molecule analysis of Ca^2+^-CaM nano-domain and Ca^2+^ saturation pathway. (A∼D) We randomly selected single CaM molecules from Fig. 5 (high Ca^2+^ injection rate) and Fig. 6 (low Ca^2+^ injection rate) and analyzed their Ca^2+^ binding kinetics both in space and in time. Panel A shows the Ca^2+^ binding state of a molecule from Fig. 5 over 2.5 ms after the start of Ca^2+^ injection. Panel B shows the trajectory of this molecule within the spine and shows its Ca^2+^ binding state in color. The red is for the fully Ca^2+^ saturated state (State N2C2 in Fig. 7A or Fig. 7E), magenta for State N1C2 and N2C1, yellow for State N1C1, N0C2 and N2C0, green for State N0C1 and N1C0, and blue for State N0C0 (see Fig. 7A and Fig. 7E for the notation). Note the direct state change between the states of the same color will never occur. Panel C and D show the same result for a randomly selected molecule from Fig. 6 over the time period of 25 ms. During this longer time period, the CaM molecule explores the entire spine head volume. The arrows in Panel A and C indicate the first N-terminal and C-terminal lobe Ca^2+^ saturation of these molecules, respectively. Panel E shows nine potential Ca^2+^ binding states of a CaM molecule and the two dominant kinetic pathways observed in Fig. 5 and 6 that lead to full Ca^2+^ saturation of CaM (compare their color with Panel A and C). (F and G) For each Ca^2+^ injection rate (Fig. 5 and 6), we plot the number of fully Ca^2+^ saturated CaM molecules up to the designated time point (x-axis). Those CaM molecules that achieved the fully Ca^2+^-saturated state via N-terminal lobe Ca^2+^ saturation first (blue) or C-lobe first (magenta) are separately depicted.

Fig. 7 presents results from studies on the Ca^2+^ saturation pathway of CaM at the single molecule level. In Fig. 7A and 7B, we randomly selected a CaM molecule from the simulation presented in Fig. 5, and analyzed its spatial location and Ca^2+^ binding state. We plot the trajectory of this molecule in the spine with different colors representing the different Ca^2+^ occupied states. The red is for the fully Ca^2+^ saturated state (State N2C2 in Fig. 7A or Fig. 7E), magenta for State N1C2 and N2C1 (three Ca^2+^ bound state), yellow for State N1C1, N0C2 and N2C0 (two Ca^2+^ bound state), green for State N0C1 and N1C0 (one Ca^2+^ bound state), and blue for State N0C0 (apo CaM) (see Fig. 7A and Fig. 7E for the notation). Note the direct state change between the states of the same color will never occur (see Fig. 7E). The choice of color for different states seems complicated but by using this strategy, we can explicitly show the state changes of a CaM molecule with a minimum number of colors.

The CaM molecule we selected for Fig. 7A and B was located relatively close to the channel at time 0 (in blue, but not clearly visible behind other colors in Fig. 7B). It went through N0C1 (green) and N1C1 (yellow) states, reached the N-terminal Ca^2+^ saturated state (N2C1, magenta), and then fully Ca^2+^ saturated (N2C2, red) near the channel (use Fig. 7A and 7E to follow these state changes). In other words, this CaM molecule follows the sequence of N-terminal lobe Ca^2+^ saturation before becoming fully Ca^2+^ saturated (indicated by the arrow in Fig. 7A). There is no C-terminal lobe Ca^2+^ saturation before the N-terminal lobe. After becoming fully Ca^2+^ saturated, the molecule started to move away from the channel but its C-terminal lobe remained Ca^2+^ saturated and stays in the N2C2 (red), N1C2 (magenta), and N0C2 (yellow) states as it explores the space close to the channel (Fig. 7B).

Fig. 7C and 7D show the single molecule analysis for the low injection rate (0.07 Ca^2+^ ions/µs). We randomly selected a CaM molecule from the simulation presented in Fig. 6 and kept track of its state change (Fig. 7C) and spatial location (Fig. 7D). This CaM molecule was located in the middle of the spine head at the beginning of the simulation and explored a large area in the spine head in N0C0 (blue) state before reaching the N0C1 (green) state. It briefly went into the N1C1 (yellow) state and returned to the N0C1 (green) state and then it reached the N0C2 (yellow) state, the Ca^2+^ saturated state of the C-terminal lobe (indicated by the arrow in Fig. 7C; also follow these state changes in Fig. 7E). After the C-terminal lobe saturation, it undergoes a rapid Ca^2+^ binding to the N-terminal lobe (at time ∼6.5 ms) via states N1C2 (magenta) to reach the fully Ca^2+^ saturated state (N2C2, red) (Fig. 7C). After Ca^2+^ is released from the fully Ca^2+^ saturated C-terminal lobe, this CaM molecule undergoes multiple state changes between N0C0 (blue), N0C1 (green), and N1C1 (yellow) states (see Fig. 7C and E).

These analyses (Fig. 7A and 7C) revealed two distinctive Ca^2+^ saturation pathways: N-terminal first pathway and C-terminal first pathway (see Fig. 7E). Fig. 7F and 7G present results that address the generality of the single examples shown in 7A and 7C. In these figures, we use the data from Fig. 5/6 and plot the number of CaM molecules that have reached the Ca^2+^ saturated state (for the first time) up to each time point (cumulative sum). We plot the number of CaM molecules who have reached saturation via N-terminal lobe saturation first (blue) and via C-terminal lobe first (magenta). At the lower Ca^2+^ injection rate, the C-terminal lobe first is the dominant pathway (Fig. 7F). At the higher Ca^2+^ injection rate, the probability of CaM reaching the fully Ca^2+^ saturated state via the N-terminal lobe first pathway is significantly increased, especially during the first 5 ms (Fig. 7G). Note it is this first ∼5 ms time period that the number of Ca^2+^ saturated N-terminal lobes exceed that of the Ca^2+^ saturated C-terminal lobe (Fig. 5A). Overall, the C-terminal lobe first pathway exists for both low and high Ca^2+^ injection rates. The Ca^2+^ saturation of CaM via the N-terminal lobe dominant pathway only becomes prominent at higher Ca^2+^ injection rates.

### Channel Distribution and its Impact on the Spatial Domain of Ca^2+^-CaM Activation

So far we have analyzed the lobe-specific Ca^2+^-CaM spatial domains using a “model” channel. The purpose of this arrangement was to systematically analyze the impact of Ca^2+^ injection rates that may underlie possible lobe-specific Ca^2+^-CaM nano-domains. We now explore the same issue under a more realistic situation. Instead of a single “model” Ca^2+^ channel, we place multiple NMDA receptors on the spine head and analyze the impact of their spatial distribution on the lobe-specific Ca^2+^-CaM nano-domain.

**Figure 8:**
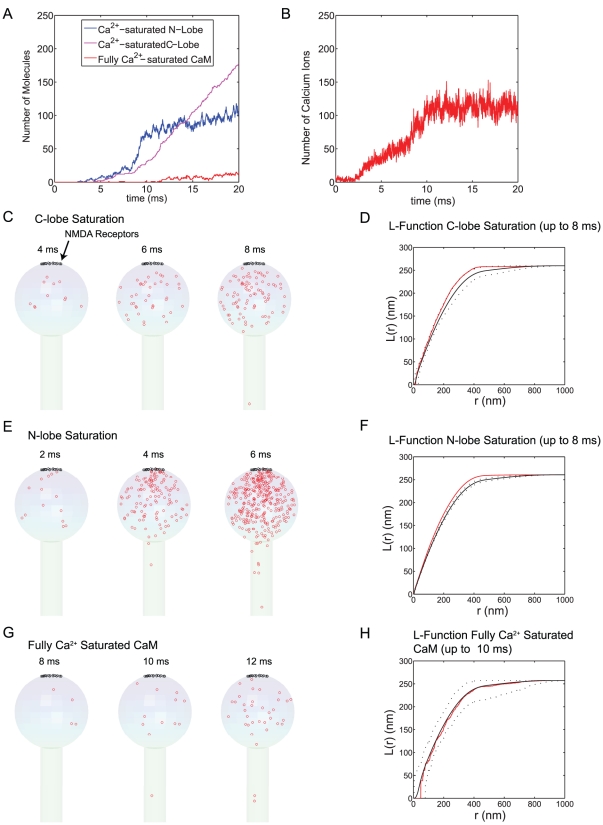
The impact of a NMDA receptor cluster dependent Ca^2+^ signal on the Ca^2+^-CaM nano-domain. Twenty NMDA receptors were placed on the top of the spine head (within 100 nm radius from the very top center of the spine head). The number of CaM molecules (1600) was the same as in Fig. 5. The population dynamics of Ca^2+^ saturated N-lobe (blue) and C-lobe (magenta) lobe are depicted along with fully Ca^2+^ saturated CaM (red; Panel A). The Ca^2+^ transient over 20 ms of simulation is also shown (Panel B). The location of Ca^2+^ saturation of C- and N- lobes of CaM and the fully Ca^2+^ saturated CaM are shown up to the designated time point (Panel C, E, and G, respectively). These results are collected from 20 simulation runs. We performed the statistical spatial point pattern analysis for the data in C, E, and G (see Results as well as Method sections for details). The mean, maximum, and minimum of Besag's L-function for complete spatial randomness were calculated (solid black line for the mean and dashed lines for max/min envelope) and compared with the L-function of the data points (up to the designated time point = 8 ms for each lobe and 10 ms for full Ca^2+^ saturated CaM, in red) (Panel D, F, H).

**Figure 9:**
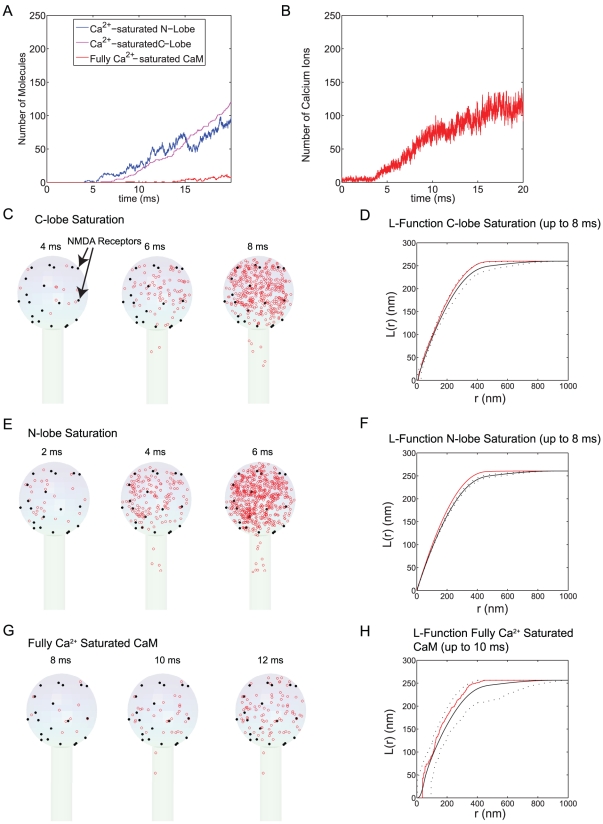
Ca^2+^-CaM nano-domain of homogenously distributed NMDA receptor dependent Ca^2+^ signal. Twenty NMDA receptors were placed randomly over the entire the spine head. The number of CaM molecules (1600) was the same as in Fig. 8. The population dynamics of Ca^2+^ saturated N-lobe (blue) and C-lobe (magenta) lobe are depicted along with fully Ca^2+^ saturated CaM (red; Panel A). The Ca^2+^ transient over 20 ms of simulation is also shown (Panel B). The location of Ca^2+^ saturation of C- and N- lobes of CaM and the fully Ca^2+^ saturated CaM are shown up to the designated time point (Panel C, E, and G, respectively). These results are collected from 20 simulation runs. We performed the statistical spatial point pattern analysis for the data in C, E, and G (see Results as well as Method sections for details). The mean, maximum, and minimum of Besag's L-function for complete spatial randomness were calculated (solid black line for the mean and dashed lines for max/min envelope) and compared with the L-function of the data points (up to the designated time point = 8 ms for each lobe and 10 ms for full Ca^2+^ saturated CaM, in red) (Panel D, F, H).

As stated earlier, NMDA receptors are the major Ca^2+^ source in CA1 spines [25]. The estimated number of NMDA receptors lie between 5∼20 [42], [43]. The number and distribution of NMDA receptor may vary from one spine to the other. To gauge the impact of the spatial localization of NMDA receptors, we decided to create two extreme cases. In Fig. 8, we placed 20 NMDA receptors in a 200 nm diameter area of the spine membrane to mimic NMDA receptors embedded in the post-synaptic density. In Fig. 9, we uniformly distributed the same number of NMDA receptors over the entire spine head. In both cases, we populated the spine volume with the same number of CaM molecules and Ca^2+^ pumps as in Fig. 5 and 6 (see Methods for more details of simulation).

In panel A and B of Fig. 8 and 9, we show the Ca^2+^ binding kinetics and free Ca^2+^ transients of single simulation runs of each case. The stochastic fluctuation (opening and closing) of NMDA receptors dictates the Ca^2+^ transient as predicted by previous work [43]. Interestingly, we could not find any significant differences between the two different distribution patterns of NMDA receptors (Fig. 8 and Fig. 9) in terms of overall Ca^2+^ (or Ca^2+^ binding to CaM) transients. To show the spatial patterns of Ca^2+^ saturation, we compiled the results of 20 simulation runs (of 20∼25 ms, for each NMDA receptor distribution pattern) and plot the locations of the Ca^2+^ saturated N- and C-lobe and fully Ca^2+^ saturated CaM as before (Fig. 8 C∼H and Fig. 9 C∼H).

For both distribution patterns of NMDA receptors, the N-terminal lobe Ca^2+^ saturation exhibits deviations from spatial randomness (Fig. 8F and Fig. 9F). In the case of NMDA receptor clusters (Fig. 8), a transient nano-domain of Ca^2+^ saturated N-terminal lobe is formed close to the receptor cluster and visible in the 2D projection of the data. In contrast, there is no detectable focus of clustering of Ca^2+^ saturated N-terminal lobe for homogenous NMDA receptor distributions (compare Fig. 8E and 9E at 4 ms). However, our methodology (Ripley's K-function/Besag's L-function) still detected a slight deviation from complete spatial randomness (Fig. 9F). This may suggest that the N-terminal lobe is still sensitive to the location of NMDA receptors but their spatial pattern of Ca^2+^ saturation was not clearly visible in the 2D projection of the data. The C-terminal lobe exhibits a minor and weak deviation from the spatial randomness for both cases. Overall, the N-terminal lobe shows a nano-domain regardless of the spatial distribution pattern of NMDA receptors.

## Discussion

### The Lobe-Specific Ca^2+^-CaM Nano-Domain and Synaptic Plasticity

We have analyzed the lobe specific spatial and temporal pattern of Ca^2+^-CaM interactions at the single molecule level in synaptic spine compartments. Ca^2+^ metabolism in neuronal spines is a dauntingly complicated process that involves nonlinear interactions between channels, pumps, CaM, and other potential Ca^2+^ binding proteins. We focused on three primary biophysical factors, Ca^2+^ binding kinetics of CaM, Ca^2+^ clearance from the spine compartment, and Ca^2+^ injection rate, and dissected the spatial pattern of Ca^2+^-CaM interactions in a stepwise manner. Our results indicate that the N-terminal lobe and the C-terminal lobe of CaM have different functions in decoding Ca^2+^ signals in space and time. The N-terminal lobe is more sensitive to the Ca^2+^ transients while the C-terminal lobe is relatively resistant to the spatial gradient of Ca^2+^. Our systematic dissection (Fig. 2∼9) strongly indicated that the Ca^2+^ binding kinetics to each lobe of CaM is the key regulatory mechanism of the spatial pattern of the Ca^2+^-CaM system. Our simulation study also identified two Ca^2+^ saturation pathways and their Ca^2+^ injection-rate dependencies: the C-terminal lobe first vs. the N-terminal lobe first pathways. The simulation results showed that the former is especially prominent with the low Ca^2+^ injection rate.

What are the implications of the lobe specific functionalities of CaM, especially for the CaM-and NMDA receptor-dependent synaptic plasticity that involves CaMKII and calcineurin? In order to understand this issue, one must pay close attention to the details of Ca^2+^-CaM-target interactions. Each lobe of CaM (as well as the entire CaM molecule) undergoes a series of conformational changes upon Ca^2+^ and/or target binding. In fact, the Ca^2+^ binding and target association are thermodynamically coupled (see [8]). Target binding increases or decreases the affinity of Ca^2+^ of CaM while Ca^2+^ binding in turn changes the binding kinetics of CaM towards its targets (see below for more discussion). The changes in the Ca^2+^ binding kinetics upon target binding (i.e., due to the different conformational states of CaM) is a critical factor that may affect the spatial profile of Ca^2+^-CaM-target activation.

Another important issue to consider is that a fraction of CaM molecules may already exist in a complex with its target even at basal Ca^2+^ concentrations. Interestingly, recent experimental and modeling work suggested that the N-terminal lobe of CaM preferentially interacts with CaMKII before the C-terminal lobe [19], [44]. In fact, these kinetic studies suggest that CaM remained bound to CaMKII for extended periods at basal Ca^2+^ concentrations via the N-terminal lobe. This mode of CaM-CaMKII interaction is different from the so-called CaM-trapping by auto-phosphorylated CaMKII (see [19] for full discussion of this issue). Once bound to CaMKII via the N-terminal lobe, the C-terminal lobe of the same CaM molecule interacts with CaMKII. When bound to CaMKII, the Ca^2+^ binding kinetics of the C-terminal lobe are accelerated by the law of detailed balance [19]. As shown in Fig. S3, CaMKII bound C- and N-terminal lobes both have faster Ca^2+^ binding kinetics (Panel A) and shorter first passage time for Ca^2+^ saturation (Panel B). The present work (Fig. 2, 5∼9) predicts that CaMKII-bound CaM may exhibit a nano-domain as observed in the target-free N-terminal lobe as long as the distribution of CaMKII is homogeneous within the spines. The latter assumption (homogenous distribution of CaMKII) may not be the case. However, recent experimental results indicated the presence of a nano-domain of CaMKII activation in CA1 spines [45]. Since CaMKII plays a key role in LTP (long-term potentiation) induction, further investigation of this CaMKII nano-domain is critical.

What if the C-terminal lobe preferentially interacts with calcineurin which underlies LTD (long-term depression) induction? Then, each of the two lobes of CaM differentially regulates these two opposing processes of synaptic plasticity. This may seem like an attractive hypothesis and in fact, our preliminary modeling study indicated that the C-terminal lobe of CaM has a higher affinity toward calcineurin than the N-terminal lobe. However, the affinity of calcineurin for CaM is extremely high [17] and as a consequence, most of the calcineurin molecules may already be bound to Ca^2+^-CaM even at the basal free Ca^2+^ concentrations in CA1 spines. On the other hand, for full activation, additional Ca^2+^ must bind the regulatory subunit (subunit B, CnB) of calcineurin [17]. If the Ca^2+^ binding kinetics of CnB is similar to that of the C-terminal lobe of CaM, one would expect a spatial and temporal pattern of calcineurin activation to be similar to the C-lobe specific Ca^2+^-CaM activation domain. Detailed experimental characterization of the Ca^2+^ binding kinetics of the “CaM-like” subunit of calcineurin (CnB) is necessary.

In CA1 pyramidal neurons, another critical factor, RC3 (neurogranin, Ng), regulates the induction of NMDA-receptor and CaM-dependent synaptic plasticity. RC3 is highly enriched in CA1 spines and is known to regulate the transition between the induction of LTP vs. LTD [46], [47]. The biochemical analysis of RC3-CaM interactions suggested that it may have an additional impact on the spatial nano-domain of Ca^2+^-CaM. RC3 binds CaM (even in the absence of Ca^2+^) and accelerates the Ca^2+^ dissociation from the C-terminal lobe thereby decreasing its affinity toward Ca^2+^ [30], [48]. The thermodynamic reciprocal interaction between Ca^2+^ binding and target interaction that we mentioned earlier may play an important role in determining the spatial dynamics of Ca^2+^-CaM-RC3 interactions. The released Ca^2+^ ion can bind the N-terminal lobe of the same or another CaM molecule. We predict that RC3 has a positive impact on the N-terminal specific Ca^2+^-CaM nano-domain and on the nano-domain of CaMKII bound CaM. In addition, RC3 is known to interact with membrane phosphatidic acid [49]. The spatial distribution of RC3 and the mobility of CaM-RC3 may have an additional significant impact of the spatial dynamics of Ca^2+^-CaM activation. Overall, genetic studies clearly suggest a critical role of RC3 in the regulation of Ca^2+^ dynamics in spines [46], [47]. Together with CaMKII, RC3 is another molecular target for future study using the particle-based Monte Carlo simulation.

### Importance of Particle-Based 3D Stochastic Simulations

The persistent existence of N-terminal lobe specific Ca^2+^-CaM nano-domain (Fig. 5∼Fig. 9) may at first seem reminiscent of the traditional view on Ca^2+^ micro-domains. However, we must point out that “Ca^2+^ domains” and “Ca^2+^-CaM domains” are, strictly speaking, different concepts. A “Ca^2+^ nano-domain” is defined by the mean distance traveled by Ca^2+^ ions before being captured by buffer (Ca^2+^ binding protein) or being extruded. Only under certain conditions, for example, when the Ca^2+^ binding rate is faster than the diffusion of Ca^2+^, are “Ca^2+^ domain” and “Ca^2+^-buffer” domain closely related in space. Clearly, the C- and N- terminal lobe specific Ca^2+^-CaM domains respond differently for the same Ca^2+^ input (Fig. 5 and 6) and the spatial profile (and size) of the C-terminal lobe domain is different from the “(free) Ca^2+^-domain”. Fig. S4 illustrates this point and shows the distributions of Ca^2+^ ions, Ca^2+^ saturated N-terminal and fully Ca^2+^ saturated CaM from a single simulation run in Fig. 5 and 6. Clearly, the size and spatial profile of these domains are not identical.

The spatial profile of the “Ca^2+^” signal ([Ca]_i_ below), in the presence of excess unsaturable mobile buffers, is given by the following equation [50]:
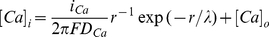
(5)where, 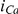 is the single channel Ca^2+^ current, 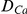 is the diffusion coefficient of Ca^2+^ (defined earlier), 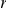 the distance from the channel, [Ca]_0_ is the bulk Ca^2+^ concentration, and 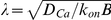 denotes the mean path length of a Ca^2+^ ion travels before being captured by buffer, *B* is the buffer concentration 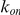 is the Ca^2+^ binding rate, and *F* is the Faraday constant. This and many other mathematical formulas have been developed (see reviews in [16]) but they are not very useful to study the spatial profile of Ca^2+^-CaM or for any other protein or buffer with multiple Ca^2+^ binding sites of different binding kinetics.

Furthermore, in a small sub-cellular compartment, like CA1 spines, the number but not the concentration of molecules is important. As an illustration, when the equation for the steady-state Ca^2+^ concentration profile is applied to an L-type Ca^2+^ channel, it predicts a sharp Ca^2+^ gradient which results in 100 µM Ca^2+^ concentration at a distance of ∼4 nm from the channel (see Fig. 1C in [51]). 100 µM of Ca^2+^ within 4 nm distance of a channel is more than sufficient to saturate the C-terminal lobe of CaM, but it corresponds to less than 1 molecule of Ca^2+^ ion, leading to a contradiction. In order to understand the spatial information flow of the Ca^2+^ signaling system in dendritic spines, one must explicitly calculate the first passage time distribution of Ca^2+^ saturation of CaM and their spatial profile using an accurate particle-based Monte Carlo algorithm and appropriate data analysis method (e.g., statistical point pattern analysis) as we did in this study.

In addition, it is important to note that the two lobes of CaM, with almost opposite impacts on Ca^2+^-CaM nano-domains, reside in the same molecule and are competing for a limited amount of Ca^2+^ as we discussed in the Results (Fig. 2). This again implies that the N- and C- terminal lobes decode Ca^2+^ signals in a different manner, and potentially serve distinct cellular functions. The current work is the first step to understand this unique functionality of CaM at the single molecule level.

### Nonlinear Control of Synaptic Ca^2+^ by CaM and by Other Factors

The Ca^2+^ transient in dendritic spines is regulated by highly nonlinear interactions between voltage-gated Ca^2+^ channels, K^+^ channels, and glutamate receptors. This important issue was recently reviewed in [25]. Clearly, Ca^2+^-activated K^+^ channels (SK channels) in hippocampal neurons shape the Ca^2+^ transients in spines and a direct coupling between voltage-gated Ca^2+^ channels and SK channels via “Ca^2+^ nano-domains” is a critical regulatory factor of spine Ca^2+^ metabolism. In addition, CaM itself regulates the activities of Ca^2+^ channels and Ca^2+^ pumps (PMCA) [52]. Without the detailed knowledge of these issues, we are not able to quantitatively address their impacts on spine Ca^2+^ dynamics. It is also difficult to make correct interpretations of pre-existing Ca^2+^ imaging experimental data (e.g., roles of pump in the diffusional coupling between dendrites and spines). For these reasons, in this study we focused on the initial rising phase of Ca^2+^ transients and therefore only studied the impacts of Ca^2+^ injection rate that are relevant for any Ca^2+^ channels.

With these limitations in mind, we repeated all simulations in Fig. 5∼9 without Ca^2+^ pumps and discovered that the resultant spatial profile of lobe specific Ca^2+^-CaM domains were similar to the results with Ca^2+^ pumps (data not shown). As long as Ca^2+^ pumps are uniformly distributed, the Ca^2+^ binding kinetics of CaM dictates the spatial and temporal pattern of the Ca^2+^-CaM interaction. We have not, however, tested spatially non-uniform distribution of Ca^2+^ pumps such as clusters of PMCA/NCX/NCXK tightly coupled to Ca^2+^ channels. This is an open area of future research.

Finally, the smooth endoplasmic reticulum (SER) is another source of Ca^2+^ that potentially influences Ca^2+^ transients in the spine. Although our simulator is fully capable of implementing SER structures and Ca^2+^ release from this source, only a small subset of dendritic spines on CA1 pyramidal neurons contain SER [53]. Furthermore, a recent study suggested a strong link between the SER containing spines and metabotropic glutamate receptor dependent synaptic depression [54] which is an interesting but different topic than the focus of the present work.

## Methods

### Mathematical Model

CaM is a bi-lobed molecule that has two Ca^2+^-binding sites within each lobe. Fig. 1A shows how this kinetic mechanism is modeled. Each lobe of CaM has three different states dependent on the number of bound Ca^2+^ ions: (apo)-CaM, (Ca^2+^)-CaM and (Ca^2+^)_2_-CaM (the horizontal arrows in Fig 1A). The resultant CaM model has nine Ca^2+^ binding states (Fig. 1C). We assume that Ca^2+^ binding to the C-lobe and N-lobe are independent and that inter-lobular cooperativity is not considered. The rate constants of Ca^2+^ binding to each lobe are taken from our previous work [8], [30]. This model is a simplification of our more elaborate model of CaM [19]. In the latter modeling scheme, Ca^2+^ association and dissociation at each Ca^2+^ binding site of CaM were explicitly modeled. Further refinement of the latter detailed model is also possible by taking into account of open (relaxed) and inactive closed (tense) states of each EF-hand of CaM as proposed by Stefan et al. [55]. We repeated the first passage time analysis in Fig. 2 using the former detailed model and confirmed that there is no qualitative difference between the detailed and simplified models. Future efforts will be made to incorporate the idea of relaxed and tense states in our simulations to specifically examine their consequences on Ca^2+^/CaM/target interactions.

The Ca^2+^ transient in the spine (head) is regulated by a highly complicated set of nested feedback loops [25]. This includes ionotropic glutamate receptors (AMPA receptors and NMDA receptors), CaV_2.3_ voltage-sensitive Ca^2+^ channels, small conductance Ca^2+^-activated K^+^ channel (SK channels), and voltage-gated Na^+^ channels. The role of voltage-gated CaV_2.3_ channels and Na channels have been largely unknown until recently [25], [56]. On the other hand, the nature of ionotropic glutamate receptors such as NMDA receptors, the major source of Ca^2+^ influx into the spine compartment, has been extensively studied in the past and we used a recently published model for our simulation (Fig. 8 and 9) [43].

The functional roles [34], [57], [58], [59] and molecular expression [60], [61], [62] of Ca^2+^ pumps have been studied; however, very limited quantitative information is available regarding the number, (intra-spine) distribution, and detailed kinetics properties of these Ca^2+^ pumps. The membrane densities of the plasma membrane Ca^2+^-ATPase (PMCA) and the Na^+^-Ca^2+^ exchanger (NCX) are 150∼300/µm^2^ of membrane and 32∼60/µm^2^ membrane, respectively [35], [63]. Since we do not have reliable data for the intra-spine distribution of these pumps, we decided to use the maximum estimated membrane densities for each pump to evaluate their impacts on Ca^2+^ dynamics (Fig. 3C). The PMCA kinetic constants are 0.2 µM K_m_ for Ca^2+^ and a turnover rate of ∼100 s^−1^ and NCX has a K_m_ of 3 µM and a turnover rate of ∼1000 s^−1^ [35]. For initial investigations we fixed the resting extrusion at 25 ions per second and 48 ions per second for PMCA and NCX, respectively [35].

The reaction scheme for the Ca^2+^ pump is similar to the one in [35]:
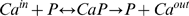
(6)where 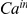, 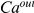, 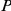 and 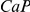 are Ca^2+^ inside the spine, extruded Ca^2+^, pump, and Ca^2+^-pump complex. PMCA hydrolyzes one ATP molecule per Ca^2+^ ion transported, i.e., exchanges one Ca^2+^ for one H^+^ (see recent reviews by Di Leva et al. [52]). NCX exchanges three Na^+^ for one Ca^2+^ and NCKX imports four Na^+^ while exporting one Ca^2+^ and one K^+^ (reviewed in [64]). Provided that we do not model the diffusions of Na^+^ or K^+^ or ATP hydrolysis, Eq. 6 captures the essential characteristics of these Ca^2+^ pumps (see Discussion for Ca^2+^-CaM dependent regulation of PMCA). Finally, we randomly incorporated Ca^2+^ leak channels so that the net flux of Ca^2+^ is 0 at rest (50 nM Ca^2+^).

The NMDA receptor kinetics was taken from previous modeling work [43]. Although our CDS simulator is fully capable of simulating glutamate release and diffusion in the synaptic cleft, this issue was not a focus of the present study. Instead, we assumed that each NMDA receptor was exposed to a constant level of glutamate as in previous modeling work [43], i.e., we stimulated the NMDA receptors for 0.1 ms with 1 mM of glutamate application and observed the subsequent Ca^2+^/CaM activation in the spine. The stochastic fluctuation of Ca^2+^ influx is then due to the stochastic kinetics of NMDA receptors.

All other numerical analyses including spatial point pattern analysis and first passage time calculation were carried out under the Matlab environment (The MathWorks, Inc., Natick, MA, USA).

### Cellular Dynamics Simulator (CDS)

The algorithmic principle of the event-driven particle-based Monte Carlo simulator (CDS) is described in [65] and the software is downloadable from our website (http://nba.uth.tmc.edu/cds). The CDS algorithm uses the discretized Brownian motion and relies on the first passage theory and event-driven simulation scheme. The overview of pre-existing particle-based Monte Carlo simulations (Smoldyn [66], GFRD [67], the coarse-grained molecular simulator described by Ridgway et al. [68], and MCell [27]) and differences between these simulator and CDS are also discussed in [65].

Under the CDS algorithm scheme, we calculate the first passage time (and probability) of molecular collisions and chemical reactions for each molecule in the simulation and create a list of all possible future events and their timing. We execute all of these molecular collisions and chemical reactions exactly as they happen one-by-one while moving all molecules simultaneously in the space. Every time we execute an event, we update the event list based on the new location or chemical status of the molecules. The time interval between two consecutive events varies from one simulation step to the other. Therefore, unlike time-driven Monte Carlo algorithms (e.g., MCell and Smoldyn), there is no fixed time step in CDS. This event-driven scheme is the only accurate way to handle molecular collisions in a crowded cellular environment. In some cases, the interval between two successive events (collision or chemical reaction) becomes long and may result in the non-Brownian motion of molecules. To avoid this situation, we add “change of direction of move” to the event list so that the direction of molecular motion is constantly randomized at least once every10 ns (the jump length of Ca^2+^ ion during this time period is smaller than the size of CaM molecule).

In the CDS simulations, the radius of gyration of CaM (2.2 nm) was used to set the size of CaM molecules. The radius of Ca^2+^ ion was set to 0.2∼0.25 nm (larger than its atomic radius) taking into account its hydration shell [69], i.e., we modeled Ca^2+^ as a solvated ion while simulating its diffusion and interactions with proteins. The diffusion coefficient (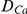) of Ca^2+^ in non-buffered cytoplasm is 200∼225 µm^2^/s (nm^2^/µs) [31].

### Spatial Point Pattern Analysis

The idea behind the Ripley's K-function, or its derivative Besag's L-function, is that if the distribution of the points is random, the number of points within a distance 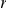 is proportional to 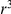 if there is no spatial boundary in the system. Suppose we have a 3D spatial distribution of 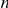 points (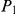
∼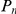) and 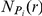 denotes the number of all points within a distance 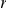 of the particular point 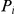 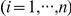. The Ripley's K-function 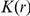 is defined by
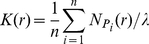
(7)where 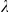 is the density of particles, the average number of particles in a unit ball [28], [41]. The expected value 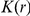 for a random Poisson distribution in infinite space is 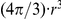. The Besag's L-function is a derivative of K-function and is defined by
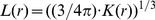
(8)so that its expected value for a random Poisson process in infinite space is 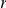 (linear). A deviation of L-function from the spatial randomness indicates a clustering or repulsion of the point distribution. We can calculate K-functions with respect to a specific point in space such as a Ca^2+^ channel (instead of 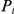
's), but in this work, we calculated (Besag's) L-function for all points in space. The latter type of L-function is important and very useful as the clustering of points (the location of Ca^2+^ saturation) can happen in the middle of the spine head when multiple channels exist or when multiple cycles of Ca^2+^ binding and unbinding to the same CaM molecule take place (Fig. 8 and 9). Our data represent an analysis of inter-point (inter-Ca^2+^-saturation point) distance distribution at all distance scales and over the entire spine compartment. The important point to note is that in a confined and complicated geometry such as dendritic spines, a simple mathematical formula of Besag's L-function for complete spatial randomness is unavailable. To overcome this constraint, we created 1000 sets of randomly distributed points in the spine of the same number of data points and then calculated the L-function for the data and for the simulated random point patterns. If the resultant L-function of the data deviates from the simulated point pattern, we can conclude that the data points are not randomly distributed.

## Supporting Information

Figure S1 Domain of Ca^2+^-CaM in a spine with a long neck. We randomly placed the same number of CaM molecules (1,600) as in Fig. 5∼6 in a spine with a longer and narrower neck (spine head radius 250 nm, neck radius 50 nm, and neck length 750 nm; see also Fig. 3A). We carried out the analyses as in Fig. 5 and 6 to test the impact of spine morphology on the spatial domain of Ca^2+^-CaM interactions. Here we show the summary of these analyses. Panel A shows the locations of Ca^2+^ saturation of C- terminal or N-terminal lobes up to the designated time point with a higher (as in Fig. 5) or lower (as in Fig. 6) Ca^2+^ injection rate. We carried out the same statistical spatial point pattern analysis as in Fig. 5∼6. Panels B and C are the Besag's L-functions for lower and higher Ca^2+^ injection rates, respectively. Each row of these panels show the L-function for the Ca^2+^-saturation of the C-terminal, N-terminal lobes and the full Ca^2+^ saturation of CaM up to the designated time point. The Besag's L-function in Panel D show the Ca^2+^ saturation of the C-terminal lobe at a low Ca^2+^ injection rate and the N-terminal lobe at a high Ca^2+^ injection rate, respectively, with the reduced diffusion coefficient of CaM (*D_CaM_* = 2 µm^2^/s). No spatial gradient is formed for the C-terminal lobe with a lower Ca^2+^ injection rate. At a higher Ca^2+^ injection rate, Ca^2+^-saturation of the N-terminal lobe exhibits a gradient around the channel. The results did not depend on the diffusion coefficient of CaM as long as the CaM molecules were randomly placed in the spine.
(2.57 MB EPS)

Figure S2 Domain of Ca^2+^-CaM in a spine with short neck. The same analyses (as in Fig. S1) were carried out in a spine with a short and wide neck (spine head radius 250 nm, neck radius 100 nm, and neck length 125 nm; see also Fig. 3A). Panel A shows the locations of Ca^2+^ saturation of N- and C- terminal lobes up to the designated time point with a lower or higher Ca^2+^ injection rate. Panels B and C are the Besag's L-functions for lower and higher Ca^2+^ injection rates, respectively. The Ca^2+^-saturation of the C-terminal, N-terminal lobes and the full Ca^2+^ saturation of CaM follow the same pattern as in Fig. 5 and 6. In summary, these results (Fig. S1∼Fig. S2) confirm that, as long as CaM is uniformly distributed in the spines, the Ca^2+^ binding kinetics is the major factor that controls the spatial domain of Ca^2+^-CaM saturation, regardless of spine morphology and the diffusion coefficient of CaM.
(1.70 MB EPS)

Figure S3 Mean first passage time of Ca^2+^ binding to CaMKII-bound CaM. (A) A kinetic diagrams showing the interactions between Ca^2+^ and each lobe of CaMKII-bound CaM. The kinetic rates of Ca^2+^ binding to each lobe of CaMKII-bound CaM are shown in Fig. 1A. Each arrow in the panel represents the corresponding rate constant. The rightward arrows indicate the Ca^2+^ association rate and the leftward arrows are the Ca^2+^ dissociation rates. These values were obtained via parameter optimization as in [19]. Note the second Ca^2+^ association rate for the C-terminal lobe is no longer slow (compare with Fig. 1A). (B) Mean first passage time (mFPT) of Ca^2+^ binding to CaMKII-bound CaM lobes are compared to target free CaM and displayed as a function of the Ca^2+^ concentration (blue and red for the N- and C-terminal lobe, respectively). Target free lobes are indicated by solid lines and CaMKII bound lobes are shown by dashed lines of the same color. The unit of first passage time (y-axis) is seconds. The range of Ca^2+^ concentrations considered is from 0.05 µM (resting level)∼12 µM (close to the peak Ca^2+^ concentration during the synaptic stimulation). The inset shows the blow up of mFPT near ∼1 µM Ca^2+^ concentrations.
(1.32 MB EPS)

Figure S4 The spatial domain of Ca^2+^ and lobe-specific Ca^2+^-CaM domain. These panels are snapshots from the simulations showing the spatial correlation between free Ca^2+^ ions with respect to Ca^2+^ saturated CaM. For illustration purpose, the sizes of molecules in the figure were artificially enlarged and are not proportional to their physical dimensions. In fact, the large light brown dots are free Ca^2+^ ions at the designated time points. Each of these panels are taken from a single simulation run used in Fig. 5 (Panel A and B, high Ca^2+^ injection rate) and Fig. 6 (Panel C and D, low Ca^2+^ injection rate). The small blue dots are the locations of (first) N-terminal lobe saturation (for each CaM molecule) taken from the same simulation. The red points are the locations of (first) full Ca^2+^ saturation of CaM up to the designated time point. Note Ca^2+^ ions are still being injected in Panel D at the designated time point but there is no significant spatial gradient of Ca^2+^ or the N-terminal lobe Ca^2+^ saturation. In Panel B (high Ca^2+^ injection rate), the (first) full Ca^2+^ saturations of CaM took place away from the channel.
(1.56 MB EPS)
